# Influenza A and Streptococcus pneumoniae Co-infection Presenting With Cardiomyopathy and Acute Kidney Injury in a Previously Healthy Adult: A Case Report

**DOI:** 10.7759/cureus.84032

**Published:** 2025-05-13

**Authors:** Brianna Tomlinson, Kobe Saldaña, Jackie G Kelley, James Kelley, Ayan Sen, Lauren B Querin, Wayne A Martini, Douglas Rappaport

**Affiliations:** 1 Emergency Medicine, Mayo Clinic Alix School of Medicine, Scottsdale, USA; 2 Emergency Medicine, University of Arizona College of Medicine, Tucson, USA; 3 Emergency Medicine, Mayo Clinic Arizona, Phoenix, USA; 4 Critical Care, Mayo Clinic Arizona, Phoenix, USA

**Keywords:** acute kidney injury, infection-associated cardiomyopathy, influenza a, mixed shock, streptococcus pneumoniae

## Abstract

Cardiomyopathy is a serious complication of systemic infections, commonly linked to viral myocarditis and sepsis-induced myocardial dysfunction. Although influenza and Streptococcus pneumoniae are independently associated with cardiovascular complications, cases involving concurrent infection with both pathogens in the absence of bacteremia remain exceedingly rare.

We describe a case of acute reversible cardiomyopathy in a previously healthy 31-year-old male presenting with shock, acute kidney injury, and left ventricular dysfunction. The patient tested positive for influenza A by reverse transcription polymerase chain reaction (RT-PCR) from a nasopharyngeal swab and Streptococcus pneumoniae via urine antigen assay, without evidence of bacteremia from negative blood cultures. Transthoracic echocardiography on admission revealed a left ventricular ejection fraction (LVEF) of 26% with biventricular dysfunction. Despite initial hemodynamic instability, the patient responded to supportive care including vasopressors, inotropes, antiviral and antibacterial therapy, and was transitioned to guideline-directed medical therapy for heart failure. By day five of hospitalization, repeat echocardiography demonstrated recovery of LVEF to 59% and resolution of organ dysfunction.

This case highlights a rare but clinically significant manifestation of infection-induced cardiomyopathy associated with concurrent influenza A and S. pneumoniae infection without bacteremia. Proposed mechanisms include direct viral cytotoxicity, influenza-enhanced translocation of pneumococci into myocardial tissue, and a synergistic immune-mediated inflammatory response. The associated acute kidney injury underscores the severity of systemic involvement. Prompt recognition, comprehensive diagnostic evaluation, and multidisciplinary management were crucial in reversing cardiac dysfunction and achieving full recovery.

## Introduction

Cardiomyopathy is a recognized and serious complication of systemic infections, most commonly resulting from viral myocarditis or sepsis-induced myocardial dysfunction associated with bacterial infections. Sepsis-induced cardiomyopathy has been linked to mortality rates as high as 70% [1], and diagnosis remains challenging due to a lack of universally accepted diagnostic criteria [2]. Viral myocarditis, although relatively uncommon with an estimated incidence of approximately 2.56%, can lead to acute or chronic cardiac dysfunction, including dilated cardiomyopathy [3]. Common viral pathogens associated with myocarditis include parvovirus B19, human herpesvirus 6, and coxsackie B viruses [4]; however, there is increasing recognition that other pathogens, such as influenza and Streptococcus pneumoniae, can also induce significant cardiac injury.

Influenza virus infection is increasingly recognized for its potential to cause serious cardiovascular complications, significantly impacting patient morbidity and mortality. Myocarditis and subsequent cardiomyopathy are severe but frequently underdiagnosed complications of influenza infection. A recent systematic review and meta-analysis highlighted elevated risks and cumulative incidence of influenza-associated cardiovascular events [3]. Similarly, Streptococcus pneumoniae, a common cause of community-acquired pneumonia, has been increasingly recognized for its capacity to induce acute cardiac injury and cardiomyopathy. Recent observational studies suggest that approximately 7-8% of patients hospitalized with pneumococcal pneumonia may develop cardiac complications. Evidence from animal and human studies highlights direct cardiotoxicity and potential long-term cardiac remodeling associated with pneumococcal infections [5,6].

Here, we present a case of mixed illness-associated cardiomyopathy in a 31-year-old patient, occurring in the setting of influenza A and Streptococcus pneumoniae infection.

## Case presentation

The patient, a 31-year-old male, presented with a nonproductive cough, dizziness, non-bloody diarrhea, and a single episode of vomiting. He had experienced an upper respiratory illness starting 10 days prior, initially improving but worsening 48 hours before presentation. Upon emergency department arrival, his blood pressure was 85/57 mmHg. He was afebrile, tachycardic at 109 bpm, with normal respiratory rate and pulse oximetry reading of 96%.

**Figure 1:**
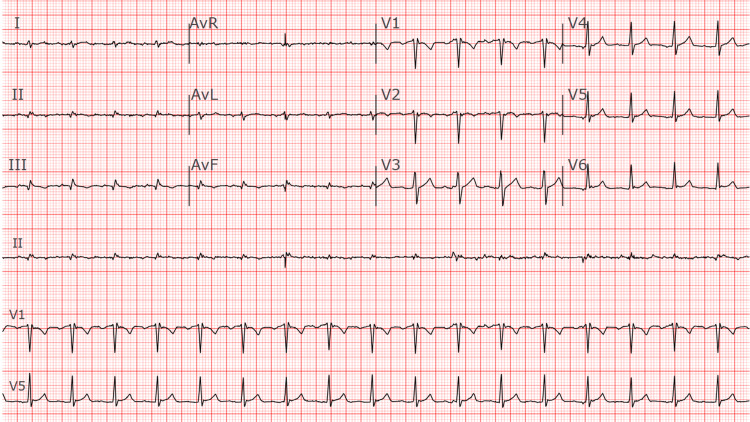
Electrocardiogram The ECG demonstrates sinus tachycardia with a heart rate of approximately 104 beats per minute. The QRS complexes are of low voltage, as evidenced by diminished amplitude in the limb and precordial leads. There is a left posterior fascicular block (LPFB), suggested by right axis deviation in the absence of other causes such as right ventricular hypertrophy or lateral myocardial infarction. No signs of acute ischemia or infarction are observed. No prior ECGs are available for comparison.

Physical examination revealed mild pallor and dry mucous membranes. Hypotension persisted despite one liter of intravenous fluids. A bedside transthoracic echocardiogram demonstrated severe left ventricular hypokinesis with an ejection fraction of 26% and moderate to severely reduced right ventricular systolic function (Video 1). The electrocardiogram showed sinus tachycardia with low voltage QRS complexes and flattened T waves (Figure 1). Laboratory tests revealed lactate at 6.6 mmol/L, leukocytosis of 23.2 (neutrophil predominance), positive influenza A RT-PCR nasal swab, and positive Streptococcus pneumoniae urine antigen. Procalcitonin was elevated at 22 ng/mL, but blood cultures were negative. He additionally had acute kidney injury (blood urea nitrogen (BUN) 26.9 mg/dL, creatinine 2.69 mg/dL), and elevated N-terminal pro B-type natriuretic peptide (NT-proBNP) (3551 pg/mL). High-sensitivity troponin levels progressively increased from initial 15 ng/L to 41 ng/L over six hours (Table 1).

**Video 1:** Initial parasternal long ultrasound. Initial parasternal long ultrasound showing ejection fraction of 26%.

**Table 1:** Laboratory Results MCV: mean corpuscular volume; RBC: red blood cells; ELXM: electronic crossmatch; POCT: point-of-care test; BUN: blood urea nitrogen; GFR: glomerular filtration rate; NT-Pro BNP: N-terminal pro B-type natriuretic peptide; TSH: thyroid-stimulating hormone; PCR: polymerase chain reaction

Test	Reference Range (if known)	Result	Flag
Hemoglobin	13.2 – 16.6 g/dL	13.5	–
Hematocrit	38.3 – 48.6 %	40.7	–
Erythrocytes	4.35 – 5.65 x10¹²/L	4.76	–
MCV	78.2 – 97.9 fL	85.5	–
RBC Distribution Width	11.8 – 14.5 %	13.2	–
Platelet Count	135 – 317 x10⁹/L	243	–
Leukocytes	3.4 – 9.6 x10⁹/L	23.2	H
Neutrophils	1.56 – 6.45 x10⁹/L	21.68	H
Lymphocytes	0.95 – 3.07 x10⁹/L	0.52	L
Monocytes	0.26 – 0.81 x10⁹/L	0.92	H
Eosinophils	0.03 – 0.48 x10⁹/L	0	L
Basophils	0.01 – 0.08 x10⁹/L	0.06	–
Nucleated RBC	/100 WBC	0	–
D-DIMER, P	≤500 ng/mL FEU	1765	H
ELXM Eligible	–	N	–
Glucose, POCT, B	70 – 140 mg/dL	132	–
Sodium, S	135 – 145 mmol/L	138	–
Potassium, S	3.6 – 5.2 mmol/L	4.3	–
Chloride, S	98 – 107 mmol/L	100	–
Bicarbonate, S	22 – 29 mmol/L	19	L
Anion Gap	7 – 15	19	H
BUN, S	8 – 24 mg/dL	26.9	H
Creatinine	0.74 – 1.35 mg/dL	2.69	H
Estimated GFR (eGFR)	≥60 mL/min/BSA	31	L
Calcium, Total, S	8.8 – 10.2 mg/dL	9.1	–
Glucose, S	70 – 140 mg/dL	137	–
Magnesium	1.7 – 2.3 mg/dL	1.5	L
Phosphorus (Inorganic), S	2.5 – 4.5 mg/dL	2.4	L
Bilirubin, Total, S	0.0 – 1.2 mg/dL	0.9	–
Bilirubin, Direct, S	0.0 – 0.3 mg/dL	0.4	H
ALT (Alanine Aminotransferase), S	7 – 55 U/L	29	–
AST (Aspartate Aminotransferase), S	8 – 48 U/L	31	–
Alkaline Phosphatase, S	40 – 129 U/L	101	–
Protein, Total, S	6.3 – 7.9 g/dL	6.1	L
Albumin, S	3.5 – 5.0 g/dL	3.8	–
Lactate	0.5 – 2.2 mmol/L	6.6	H
Lipase, S	13 – 60 U/L	29	–
Troponin T, Baseline, 5th gen	≤15 ng/L	15	Upper normal
Creatine Kinase (CK), S	30 – 200 U/L	142	–
NT-Pro BNP	Age/sex dependent	3551	H
TSH, Sensitive	0.3 – 4.2 mIU/L	3.3	–
Procalcitonin, S	<0.10 ng/mL	22	H
Acetaminophen, S	<10 µg/mL	<5	–
Salicylate, S	<30 mg/dL	<3.0	–
Influenza A, PCR	–	Detected	!
Influenza B, PCR	–	Undetected	–
SARS-CoV-2, PCR	–	Undetected	–

Critical care medicine was consulted for ICU admission. Therapy included inotropic support with dobutamine, vasopressor support with norepinephrine, and intravenous solumedrol 60 mg as stress-dose steroids following septic shock protocol due to persistent hypotension despite adequate fluid resuscitation. Additionally, the patient received oseltamivir, ceftriaxone (1 g daily for seven days), and azithromycin (500 mg daily for three days). Pulmonary artery catheterization findings (cardiac index 2.0 L/min/m², pulmonary capillary wedge pressure 18 mmHg, systemic vascular resistance 1450 dyn·s/cm⁵) confirmed mixed cardiogenic and septic shock.

Over the subsequent two days, vasopressors and inotropes were successfully weaned with normalization of lactate. Concurrently, acute kidney injury resolved without renal replacement therapy, evidenced by normalization of creatinine and BUN and absence of intrinsic renal disease on imaging and urinalysis, suggesting acute kidney injury (AKI) secondary to systemic illness and shock. On day five, echocardiography showed recovery of ejection fraction to 59% and resolution of biventricular dysfunction (Video 2), with NT-Pro BNP improving to 498 pg/mL. The patient was discharged on losartan and metoprolol with planned cardiology follow-up.

**Video 2:** Recovered Parasternal Ultrasound Parasternal Ultrasound showing recovery of 59% ejection fraction.

## Discussion

We describe a unique case of both influenza and S. pneumoniae-associated cardiomyopathy complicated by mixed shock and acute kidney injury, without bacteremia. Although it is established that influenza virus is associated with increased risk for pneumococcal infection, as demonstrated by increased rates of hospitalizations for pneumococcal pneumonia during the 2009 influenza pandemic [7], recent studies help to demonstrate possible mechanisms. In addition to general weakening of the immune system due to influenza infection, a quantitative proteomic and molecular study demonstrated that pandemic influenza A virus is associated with increased translocation of S. pneumoniae into cardiac tissue [8]. This causes proteomic changes allowing for increased pathogenicity through adhesion factors and cytotoxicity, increasing the risk for severe cardiac and extrapulmonary complications [8]. In the setting of pneumococcal pneumonia alone, the degree of cardiac injury is dependent on high-grade bacteremia and its pathogenicity can be related to the specific strain and mechanisms may include microlesions of cardiac myocyte tissue [9]. However, the ability for influenza to increase the translocation of S. pneumoniae directly into cardiac myocytes could explain the pathophysiology in our patient’s case in the setting of negative blood cultures.

Our patient was also found to have an acute kidney injury of an unknown duration which did reverse with treatment after several days. This could indicate a prerenal azotemia related to initial cardiogenic type shock and/or hypovolemia; however, we also hypothesize that the patient’s infection itself could have contributed to kidney injury as influenza has been shown to infect and replicate within renal cells with the presence of acute kidney injury indicating severe infection and is associated with increased mortality [10]. Pneumococcus can also cause an increased risk for acute kidney injury [11]. Overall, the presence of an acute kidney injury in the setting of these infections indicates risk for poorer outcomes and should be closely monitored and treated by the physician.

The prognosis of infection-induced cardiomyopathy is affected by several factors, including the severity of cardiac dysfunction, the presence of sepsis or associated end-organ failure, the patient's age (>65), immunocompromised status, and underlying chronic comorbidities. A recent meta-analysis revealed that sepsis-induced cardiomyopathy (SICM) is found in approximately 20% of sepsis patients, with possible reversible myocardial dysfunction within seven to 10 days of appropriate management. While SICM was not consistently associated with increased short-term mortality in the overall analysis, subgroup analyses demonstrated significant associations with higher mortality [12]. These findings suggest that the prognosis of SICM may vary depending on study quality, diagnostic criteria, regional differences, and patient factors such as sepsis severity and comorbid conditions. This highlights the importance of early recognition, diagnosis, and treatment to increase the likelihood of reversing myocardial damage.

Cardiac biomarkers have become valuable tools for risk stratification and prognostication in infection-induced cardiomyopathy. In a prospective cohort study of hospitalized patients with influenza, elevated levels of high-sensitivity troponin T (hs-TnT) were significantly associated with increased mortality, highlighting the ability of myocardial injury markers to serve as a useful prognostic tool in this population [13]. Similarly, another study confirmed the role of hs-TnT as a predictive biomarker for adverse outcomes in influenza-related cardiac complications [14]. When evaluating community-acquired pneumonia, particularly infections caused by S. pneumoniae, elevated levels of cardiac biomarkers such as troponin and BNP have also been shown to correlate with increased cardiovascular risk and mortality, even among patients without pre-existing heart disease [15,16]. These findings demonstrate the importance of monitoring cardiac biomarkers in patients with influenza and/or S. pneumoniae-induced cardiomyopathy to encourage early intervention with adequate management and reduce further permanent damage.

The severity of concurrent infection and organ dysfunction may suggest a more complex prognosis in our patient. The infection with both influenza and S. pneumoniae is associated with a particularly poor prognosis due to the exacerbated myocardial injury, increased systemic inflammation, and accelerated cardiac decompensation. These pathogens are independently linked to significant cardiovascular complications, and the presence of both may enhance the risk of myocardial damage and hemodynamic instability which future research may further elucidate and quantify this relationship.

## Conclusions

This case illustrates a rare occurrence of acute cardiomyopathy in a 31-year-old without known cardiac conditions, who was found to have influenza and S. pneumoniae but without evidence of bacteremia, indicating a complex and synergistic pathogenicity between these two organisms. Further, this case was complicated by mixed shock and acute kidney injury. This patient was successfully treated with supportive care, antiviral therapy, antibacterial therapy, and heart failure guideline-directed medical therapy. This case emphasizes the importance of recognizing cardiac complications in young, relatively healthy patients with respiratory illness and how they can occur even without evidence of bacteremia. Physicians should continue to be cognizant of these rare but serious complications of common pathogens, and consideration of early bedside echocardiogram.
